# Advances in Timed Artificial Insemination: Integrating Omics Technologies for Enhanced Reproductive Efficiency in Dairy Cattle

**DOI:** 10.3390/ani15060816

**Published:** 2025-03-13

**Authors:** Jesse Oluwaseun Ayantoye, Hubdar Ali Kolachi, Xiaomeng Zhang, Muhammad Shahzad, Omaima Mohamed Tawfik Kandil, Pengcheng Wan, Xueming Zhao

**Affiliations:** 1 Institute of Animal Sciences (IAS), Chinese Academy of Agricultural Sciences (CAAS), No. 2 Yuanmingyuan Western Road, Haidian District, Beijing 100193, China; 2023y90100045@caas.cn (J.O.A.);; 2 Department of Animal Reproduction and Artificial Insemination, Veterinary Research Institute, National Research Centre, Tahrir Street, Dokki, Cairo 12622, Egypt; 3 State Key Laboratory of Sheep Genetic Improvement and Healthy Breeding, Institute of Animal Husbandry and Veterinary Sciences, Xinjiang Academy of Agricultural and Reclamation Sciences, Shihezi 832000, China

**Keywords:** timed artificial insemination, GnRH, estradiol/progesterone, dairy cattle, reproductive efficiency, omics technologies

## Abstract

Reproductive efficiency is crucial for dairy farm productivity, but achieving consistent fertility remains challenging. TAI improves pregnancy rates by synchronizing ovulation and enabling precision breeding. Despite the advancements in hormone synchronization protocols, outcomes vary due to genetic, physiological, and metabolic differences among cows. This review examines current TAI protocols, including gonadotropin-releasing hormone (GnRH)-based and estradiol/progesterone (P4)-based synchronization methods, emphasizing their effectiveness and practical limitations. We also examined how to integrate emerging omics technologies, such as genomics, transcriptomics, proteomics, and metabolomics, into TAI protocols, marking a significant shift in reproductive management. These tools unveil molecular biomarkers that enable the precise tailoring of TAI protocols to the genetic, metabolic, and physiological profiles of individual animals, addressing challenges in variable fertility responses. Key factors influencing TAI success, such as animal health, environment, and insemination timing, are explored. This review identifies gaps in the existing literature, such as the integrated omics methodologies and data integration across platforms, and proposes a framework for future research to refine TAI protocols to address genetic variability and apply omics technologies to identify validated biomarkers for early pregnancy detection, which will significantly enhance the practical impact of TAI. Future directions highlight the need for interdisciplinary approaches combining molecular insights with robust on-farm applications to improve fertility outcomes and reduce reliance on blanket synchronization methods. By combining traditional synchronization methods with cutting-edge molecular tools, TAI offers promising opportunities for improving reproductive efficiency and sustainability in dairy farming.

## Simple Summary

Reproductive efficiency in dairy cattle is essential for farm productivity, but achieving consistent fertility remains a great challenge. Timed Artificial Insemination (TAI) is a technique used to improve pregnancy rates by synchronizing ovulation, yet outcomes vary due to genetic, metabolic, and environmental factors. This study reviews the effectiveness of current TAI protocols, including hormone-based synchronization methods, and explores how advanced molecular technologies such as genomics, proteomics, and metabolomics can enhance reproductive success. By identifying biomarkers linked to fertility, these technologies allow for more precise breeding strategies tailored to individual animals. Factors like cow health, nutrition, and environmental conditions are critical to TAI’s success. This study highlights gaps in research, including the need to integrate molecular data to improve fertility predictions. Future advancements in TAI, combining traditional methods with cutting-edge biological insights, could lead to better pregnancy rates, reduced hormone use, and improved sustainability in dairy farming. These innovations will help farmers optimize breeding programs, reduce economic losses due to infertility, and ensure a more efficient and productive dairy industry.

## 1. Introduction

Reproductive efficiency is fundamental to sustaining productivity in dairy cattle operations, as it directly affects milk production and overall herd profitability [1,2]. Recent studies have emphasized the benefits of optimizing reproductive performance by improving the efficiency of artificial insemination (AI), increasing pregnancy rates, reducing the use of exogenous reproductive hormones, and ultimately enhancing the overall economic returns for dairy operations without significant impacts on reproductive outcomes throughout lactation [3]. For example, Sitko et al. (2023) [4] highlighted the economic importance of reproductive management in dairy farming by showing how optimized reproductive performance through calving interval improvements maximizes milk incomes and ultimately links reproductive strategies to economic success. Similarly, Wicaksono et al. (2024) [5] assessed the impact of systematic hormone-based reproductive management programs compared with traditional methods reliant on veterinary diagnosis. They highlighted that improving reproductive efficiency, particularly by reducing calving intervals, directly boosts milk production, increases profitability, and lowers culling rates due to fertility issues. Thus, optimizing efficient reproductive strategies enhances herd productivity and ensures long-term sustainability and profitability for dairy farming operations. However, reproductive technologies, including TAI and estrous synchronization (ES), are essential to enhancing reproductive outcomes and securing the long-term sustainability of dairy farming [6].

TAI has emerged as a critical tool in improving pregnancy rates by synchronizing ovulation, allowing for more controlled and precise breeding practices in dairy cattle [7]. TAI protocols like Ovsynch, Presynch or Double-Ovsynch, and Cosynch are cost-effective strategies for enhancing reproduction in lactating dairy cows [8]. For instance, Stangaferro et al. (2018) [9] compared Double-Ovsynch and Presynch–Ovsynch for first-service AI, defining Day 0 as calving. Cows in the Double-Ovsynch protocol were inseminated at 60 ± 3 days in milk (DIM), while cows in Presynch–Ovsynch were inseminated at 88 ± 3 DIM. The study found that the time to pregnancy was similar for cows inseminated at 60 DIM with both protocols. However, delaying insemination to 88 DIM resulted in delayed pregnancy, indicating that shorter voluntary waiting periods are more effective and profitable. Similarly, Brozos et al. (2021) [10] compared two TAI protocols and a modified G7G protocol, a 7-day GnRH-based protocol followed by heat detection, for first-service AI in dairy cows. The 5-day and 7-day Ovsynch protocols had similar pregnancy rates (33.8% and 35.2%, respectively), while the modified G7G protocol was more effective (49.1%). The 5-day protocol offered cost savings with shorter treatment and reduced labor. In contrast to general reproductive strategies, TAI enhances the precision and control of breeding, improving pregnancy rates and operational efficiency in dairy cattle.

Unlike traditional estrous detection, which is limited by subtle signs and time constraints, TAI offers the advantage of fixed breeding schedules, enhancing the efficiency of AI [11]. For example, studies show that combining TAI with traditional estrous detection methods further enhances overall reproductive performance in dairy herds, maximizing pregnancy success rates and optimizing resource use [12]. Similarly, a study on a targeted reproductive management (TRM) program in lactating Holsteins found that prioritizing AI based on detected estrus significantly improved reproductive performance, especially for cows identified in estrus during the voluntary waiting period [13]. These findings demonstrate that integrating TAI with focused estrous detection strategies can maximize reproductive efficiency, improve herd productivity, and enhance the profitability of dairy operations. Beyond enhancing the efficiency of breeding schedules, TAI also offers strategic advantages in genetic improvement and herd management, both of which contribute to the profitability and sustainability of dairy operations.

TAI improves genetic selection, optimizes herd management, and enhances profitability by advancing genetics and shortening the calving–conception interval [14,15]. For example, studies have shown a strong correlation between the genomic daughter pregnancy rate (GDPR), a genetic index used to estimate a cow’s fertility potential and reproductive success, with higher GDPRs associated with increased pregnancy rates, shorter conception times, fewer services per conception, and reduced pregnancy losses. This makes GDPR a valuable tool for improving herd reproductive efficiency and selective breeding strategies, particularly for first and subsequent inseminations [16,17]. Furthermore, integrating GDPR with TAI accelerates genetic selection, enhances synchronization efficiency, and improves fertility management and overall herd profitability. In addition, TAI also improves disease management and breeding efficiency [18,19]. In a study by Pascottini et al. (2017) [20], cytotape (CT), a diagnostic tool that allows for endometrial sampling during AI, was used to effectively diagnose cytological endometritis (CYTO) in dairy cows. With a prevalence of 27.8%, CYTO negatively impacts conception rates, but early CYTO detection through CT enables targeted treatments, improving reproductive management and overall herd health. This highlights the role of AI and TAI in enhancing disease management, breeding efficiency, and herd profitability. Cytotape provides a standardized method for diagnosing CYTO during AI, offering a practical approach to improve disease management and reproductive outcomes in dairy farming.

Additionally, recent advances in hormone synchronization protocols, such as GnRH-based and estradiol/P4-based methods, have shown varying degrees of success across different breeds [21,22] and management systems [23], offering tailored strategies to improve reproductive outcomes. For example, Pereira et al. (2017) [24] compared two protocols for increasing circulating P4 in lactating dairy cows, finding no significant difference in pregnancy rates per AI (P/AI). However, the two controlled internal drug release (CIDR) protocols consistently elevated P4 levels, benefiting reproductive performance, particularly in cows with an elevated body temperature (rectal temperature ≥ 39.1 °C). Similarly, Vazquez Belandria et al. (2023) [23] evaluated three reproductive strategies: combining estrous detection with Ovsynch, estrous detection with PRIDsynch, and TAI following a Double-Ovsynch protocol (DO), finding no significant differences in P/AI. However, primiparous cows and those with better body condition score (BCS) had higher fertility, emphasizing the role of health and nutrition in reproductive success. These findings stress the need to integrate hormone protocols with proper cow management to enhance fertility in diverse dairy systems. Moreover, while hormone synchronization improves reproduction, integrating molecular technologies enhances precision by tailoring protocols to individual cows, enabling more effective, personalized management.

However, despite the widespread use of TAI to improve herd pregnancy rates [25], reduce the interval from calving to the first service [26], and reduce inter-breeding intervals [27], significant gaps remain in understanding how emerging technologies like genomics, transcriptomics, proteomics, and metabolomics can further optimize hormone synchronization protocols for improved reproductive outcomes [28]. Omics technologies can revolutionize TAI by enabling precise, targeted reproductive strategies based on individual cows’ biology. A study by Sitko et al. (2024) [29] demonstrated how genomic merit can classify cows into fertility groups, allowing for targeted breeding strategies. Similarly, identifying endometrial DNA methylation patterns associated with pregnancy outcomes suggests that epigenomic markers can be predictive tools for fertility [30]. Therefore, integrating molecular biomarkers into TAI protocols can enhance fertility outcomes across diverse environmental and genetic conditions, addressing critical challenges in dairy cattle management. This review critically evaluates current TAI protocols in dairy cattle and explores the integration of omics technologies into hormonal synchronization to enhance reproductive efficiency in dairy cattle. We highlight the latest advancements in reproductive management and identify opportunities for future research to improve TAI outcomes through precise reproductive management. Integrating traditional synchronization methods with cutting-edge molecular insights can revolutionize TAI practices, improve fertility outcomes, enhance genetic selection, and support sustainable dairy farming systems.

## 2. Estrous Synchronization Protocols of TAI in Dairy Cattle

Estrous synchronization involves manipulating or regulating the estrous cycle to facilitate breeding at a synchronized time, thus improving the efficiency of AI and ensuring timely calving [31]. Moreover, synchronizing the estrous cycle can improve breeding outcomes, such as calf uniformity and AI capabilities, and shorten the calving season. In addition, maximizing time, labor, and financial resources can be achieved by effectively managing synchronization [32]. Recent studies have illustrated the effectiveness of hormonal estrous synchronization protocols, particularly when comparing single and double prostaglandin injections (PGF2α). Masho et al. (2024) [33] reported that double injections of PGF2α resulted in a 100% estrous response and a higher conception rate (50%) compared with single injections of PGF2α (48.1%). Similarly, a study by Haile et al. (2023) [34] found that double injections of PGF2α resulted in a 100% estrous response, whereas single injections achieved a 90.8% estrous response. These findings reinforce the value of estrous synchronization protocols, particularly double-injection methods, in achieving timely, efficient breeding outcomes that ultimately boost productivity and economic returns in dairy operations. However, an in-depth understanding of the hormonal mechanisms underlying estrous synchronization could facilitate the development of more efficient and tailored reproductive management strategies, addressing the variability in responses observed in dairy cattle and achieving optimal reproductive responses.

Effective reproductive management in dairy cattle requires a comprehensive understanding of the estrous cycle, especially when utilizing synchronization techniques. The estrous cycle typically lasts approximately 21 days and comprises four phases: proestrus, estrus, metestrus, and diestrus [35]. This cycle is regulated by a complex interplay of hormones, including GnRH, luteinizing hormone (LH), and follicle-stimulating hormone (FSH), which coordinate follicular development, ovulation, and corpus luteum (CL) formation [36,37]. Successful estrous synchronization protocols in dairy cattle exploit this hormonal interplay by manipulating these key hormones to induce ovulation predictably [33,38]. A PGF2α, such as cloprostenol, is commonly used to regress the CL, initiating a new follicular wave and ultimately triggering ovulation [39]. However, the timing of PGF2α administration is critical, as it must occur during the luteal phase when the CL is functional [40,41].

GnRH analogs, such as buserelin or gonadorelin, can be used to stimulate LH release, further enhancing the synchronization of ovulation [36,42]. GnRH is often administered alongside prostaglandins in estrous synchronization protocols to enhance the precision and predictability of ovulation timing [36,40]. PGF_2_α induces luteolysis, enabling follicular recruitment, while GnRH triggers LH release, promoting ovulation. This sequential hormone administration optimizes follicular wave emergence, ensuring a synchronized ovulatory response [36]. Rantala and Taponen (2015) [43] further highlighted that the estrous cycle stage at the time of hormone administration can influence the synchronization outcome, emphasizing the importance of precise timing in these protocols to minimize cycle variability and maximize fertility rates.

Beyond hormonal regulation, several management and physiological factors, including the cows’ nutritional status, BCS, health, and management practices, affect the effectiveness of any synchronization protocol [44,45]. A comprehensive approach that carefully considers these factors and the precise timing and selection of hormonal treatments is crucial for maximizing reproductive efficiency in dairy operations [46]. Collectively, these hormonal interactions promote the growth and maturation of ovarian follicles, initiate ovulation, and support CL formation, all of which are crucial steps for sustaining pregnancy [47]. Understanding these processes offers substantial opportunities to optimize reproductive timing, improving fertility management and herd productivity.

The successful implementation of TAI heavily relies on the synchronization of the estrous cycle, allowing for the precise timing of insemination relative to ovulation [48,49]. Several hormonal protocols have been developed to optimize this process, particularly those focusing on controlling the luteal phase and inducing ovulation [50,51,52]. The two primary types of protocols used in dairy cattle are GnRH-based protocols and estradiol/P4-based protocols.

### 2.1. Gonadotropin-Releasing Hormone (GnRH)-Based Protocols

**Figure 1:**
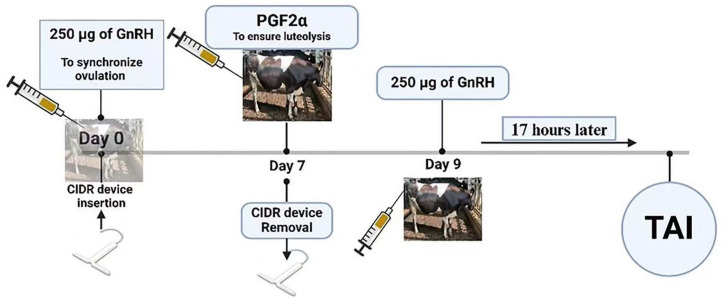
Gonadotropin-releasing hormone (GnRH)-based protocols.

GnRH-based protocols involve a series of hormonal interventions designed to synchronize ovulation and optimize AI timing. Typically, these protocols involve inserting a CIDR device and administering gonadorelin on Day 0 (Day 0 as the day of the first GnRH treatment and CIDR insertion), followed by PGF2α on Day 7, CIDR removal, and a final gonadorelin injection with TAI on Day 9 or Day 10, as shown in Figure 1 [53]. These protocols involve administering GnRH to induce ovulation and synchronize the follicular wave, followed by PGF2α to regress the CL and induce estrus [54]. Recent studies have examined modifications of the protocol to improve reproductive outcomes. Leão et al. (2024) [55] evaluated the impact of administering a different dose of gonadorelin (GnRH) in the Resynch-25 protocol for lactating Holstein cows, finding that a 200 µg dose enhanced ovulatory responses but did not significantly improve P/AI compared with a 100 µg dose. Cows without a functional CL at the time of GnRH administration showed better synchronization and higher P/AI rates. Similarly, another study assessed delaying PGF2α administration from Day 7 to Day 8 in the standard 7-day Ovsynch (GnRH-based) protocol, resulting in improved luteolysis and synchronization rates among lactating Holstein cows and significantly benefiting primiparous cows and those with lower BCS [56]. These findings highlighted the essential roles of GnRH and PGF2α in synchronizing ovulation and managing the estrous cycle, reinforcing the effectiveness of GnRH-based protocols to optimize fertility outcomes in dairy cows. However, further hormonal modifications to GnRH-based protocols have been explored to enhance fertility outcomes, especially in cows with irregular cycles or low BCS.

GnRH-based protocols, particularly the Ovsynch protocol, have been shown to improve ovulation rates and pregnancy per TAI (P/TAI) in dairy cows with regular cyclicity [57,58]. However, in cows with irregular estrous cycles or anestrus, modifications to the standard GnRH-based protocol, such as the inclusion of additional hormone treatments, e.g., equine chorionic gonadotropin (eCG) [59] or a second dose of PGF2α [22], have been suggested to improve pregnancy outcomes. Recent studies have demonstrated that adding eCG enhances follicular development and the synchronization of estrus. In contrast, adding a second PGF2α dose ensures complete luteolysis and enhances fertility outcomes in cows undergoing TAI. For instance, Randi et al. (2018) [60] investigated the impact of eCG on reproductive performance in seasonal-calving dairy cows undergoing a P4-based TAI program, finding that while synchronization improved reproductive metrics, eCG did not significantly enhance overall pregnancy rates, indicating variability in responses to eCG among cows. Similarly, Funakoshi et al. (2024) [61] explored the effects of eCG treatment during TAI under heat-stress conditions. The results showed that eCG administration significantly improved P/AI and elevated plasma P4 levels compared with the control group. However, the study indicated the limitations of its relatively small sample size, specific environmental factors, and the occurrence of multiple births, which may complicate application and management.

In addition, Liu et al. (2018) [62] focused on ovulatory response in dairy cows following luteolysis induced by two low doses of PGF2α, finding that GnRH administration advanced ovulation timing and improved synchronization; however, a small sample size and potential environmental impacts limited the applicability of these findings. Bahrami et al. (2021) [63] examined a heat-synch protocol using two PGF2α doses to improve conception rates in Holstein cows and found that shortening the synchronization interval increased the conception rate. However, variability due to individual cow characteristics limited uniformity, underscoring the importance of adapting protocols to herd management conditions. A study by Say et al. (2016) [22] focused on the fertility of Holstein heifers after two doses of PGF2α in a 5-day CO-Synch protocol, discovering that administering two doses of PGF2α at 6 h intervals significantly improved AI pregnancy rates compared with a single dose or concurrent doses; however, it lacked an exploration of the biological mechanisms behind these results. Research conducted by Stevenson et al. (2018) [64] on prostaglandin administration in dairy cows revealed that while both dosing strategies were effective, a single larger dose was less effective in a 5-day Ovsynch program, indicating a need for the further understanding of the underlying mechanisms and environmental factors affecting fertility. Lastly, Nowicki et al. (2019) [65] assessed the impact of a second PGF2α treatment during Ovsynch, concluding that it did not significantly improve overall fertility rates but reduced pregnancy loss. This highlights the necessity for alternative strategies to enhance reproductive outcomes in dairy cattle. In conclusion, while GnRH-based protocols and their modifications provide valuable tools for managing reproduction, further refinement is essential to optimize fertility outcomes across diverse herd management conditions.

### 2.2. Estradiol/Progesterone (P4)-Based Protocols

**Figure 2:**
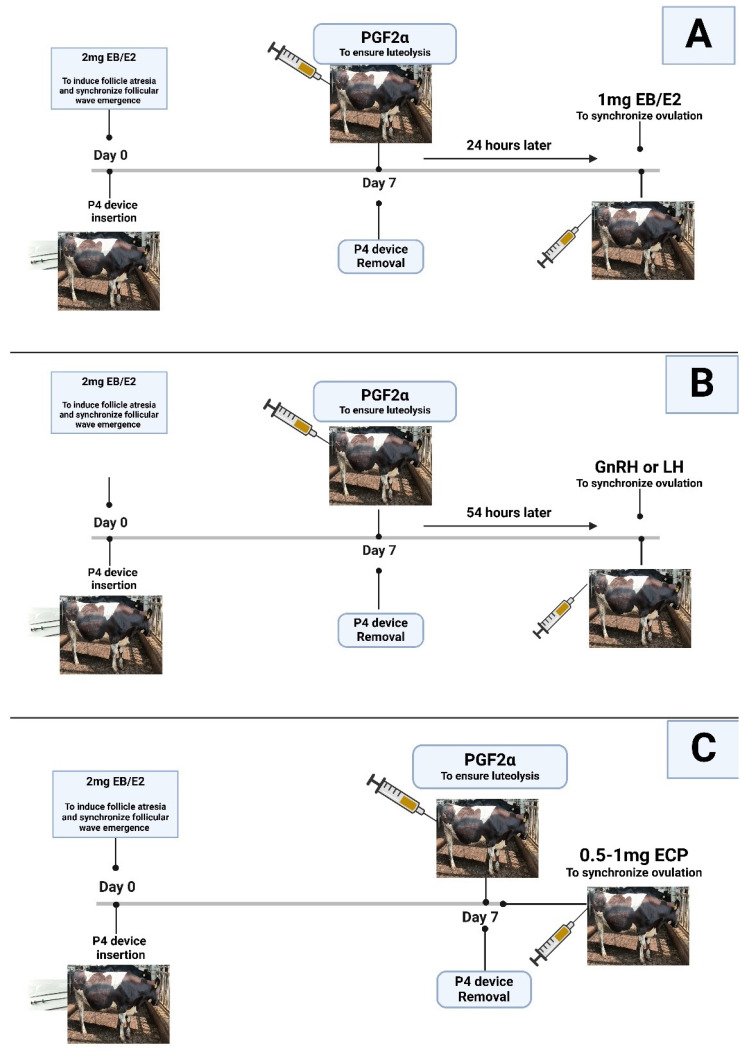
Estradiol/P4-based protocols of TAI. (**A**) 1 mg of EB/E2 24 h after PGF2α injection, (**B**) GnRH 54 h after PGF2α injection, and (**C**) PGF2α and ECP administered on Day 7 after removing the P4 device.

Estradiol and P4 treatments involve the insertion of a P4-releasing device, with estradiol benzoate (EB) or 17β-estradiol (E2) administered on a randomly selected day (Day 0) of the cycle to induce follicular atresia and synchronize the initiation of follicular waves. To facilitate luteolysis, a PGF2α injection is given at the time of P4 device removal, which occurs on Days 7, 8, or 9. Following the removal of the P4 device, ovulation synchronization is achieved by administering either EB or E2 after 24 h, GnRH after 54 h, or estradiol cypionate (ECP) during P4 device removal, as shown in Figure 2 [66]. Estradiol treatment is used to induce ovulation by promoting follicular regression and ensuring synchronized follicular wave emergence regardless of the follicular development stage at the time of treatment [67]. The effectiveness of estradiol/P4 protocols in enhancing reproductive outcomes is well documented. Tschopp and Bo (2022) [68] investigated the impact of estrous expression and GnRH administration on pregnancy rates following AI in lactating dairy cows treated with an estradiol/P4-based synchronization protocol. The study found that cows exhibiting estrus by 48 h after P4 device removal had higher P/AI rates when inseminated at that time, while those not in estrus benefited from GnRH administration and delayed insemination at 60 h, leading to the improved synchronization of ovulation and increased pregnancy rates. Similarly, Madureira et al. (2019) [69] examined how the intensity of estrus, influenced by estradiol and P4 treatments, affects fertility in dairy cows. The results demonstrated that higher estrus intensity correlates with improved fertility rates, emphasizing the importance of effective synchronization protocols. However, the study’s limitations included variability in estrous response among the cows, an insufficient exploration of biological mechanisms behind GnRH’s effects on ovulation timing, and the potential influence of external factors on fertility. These findings emphasize that estradiol and P4-based synchronization protocols enhance reproductive success. However, challenges with estrous variability and limitations in understanding hormonal influences suggest the need for further optimization in dairy cattle fertility management.

Estradiol/P4-based protocols are effective in inducing cyclicity in anestrus cows, improving pregnancy rates in non-cycling dairy cattle [70]. Additionally, these protocols offer flexibility, particularly in synchronizing estrous across large herds, making them ideal for farms with high labor demands or variable cow health statuses [71]. In another study, Consentini et al. (2022) [72] investigated the effectiveness of estradiol and P4 protocols combined with GnRH, inducing cyclicity in lactating dairy cows and enhancing pregnancy rates in non-cycling dairy cattle. The results demonstrated that these hormonal treatments significantly improve estrous synchronization and fertility, making them particularly beneficial for large and high labor demands. However, the study’s focus on specific conditions may limit its applicability across diverse herd management practices. In another study, Bandai et al. (2020) [73] evaluated a short-term TAI protocol using EB in lactating dairy cows. The results revealed that EB led to higher pregnancy rates than GnRH, demonstrating its effectiveness for synchronizing estrous in large herds. Nonetheless, the study did not explore long-term effects or variations in cow health statuses, indicating a need for additional research in these areas. Similarly, Allahyari et al. (2023) [21] evaluated the effectiveness of replacing the first GnRH injection with estradiol in the Double-Ovsynch protocol, finding that the estradiol-based approach significantly improved pregnancy rates, particularly in cows with a CL, thus demonstrating its efficacy in inducing cyclicity in anestrus cows. In conclusion, these hormonal protocols enhance pregnancy rates by promoting estrous synchronization, inducing cyclicity, and supporting luteal and follicular function, making them particularly advantageous for managing non-cycling dairy cows in high-demand herd environments.

### 2.3. Comparison of Protocols and Practical Considerations

GnRH-based and estradiol/P4-based protocols have advantages and limitations depending on herd management practices, breed types, and environmental conditions. GnRH-based protocols in dairy cattle are particularly effective in enhancing ovarian response and increasing pregnancy rates, especially in high-producing herds, due to their ability to stimulate LH release while also providing flexibility in timing that allows for adaptation to different reproductive management strategies [74,75]. In contrast, EB-based protocols offer benefits such as effective estrous synchronization and the induction of follicular wave emergence, and they are generally simple and less time-sensitive to administer than GnRH, which requires precise timing for maximum effectiveness [76]. However, the effectiveness of GnRH can be sensitive to the timing of administration relative to the estrous cycle and the physiological state of the cows, leading to potential variability in responses [76]. Estradiol/P4-based protocols, on the other hand, come with notable limitations, such as the risk of inducing luteolysis, which may negatively impact fertility if not carefully managed. Additionally, when used alone, EB-based protocols may result in lower pregnancy rates compared with GnRH-based protocols, emphasizing the need for careful consideration in their application [66]. Therefore, maximizing reproductive success in dairy cattle relies on applying GnRH-based or estradiol/P4-based protocols tailored to improve synchronization efficiency and fertility outcomes according to herd physiology and management demands. Table 1 below compares the P/TAI of critical protocols, highlighting the success rates of different synchronization methods across various studies.

As research continues, there is growing interest in refining these protocols by integrating molecular biomarkers to predict the optimal time for insemination. This approach could enhance precision in reproductive management, especially in diverse herd and environmental contexts. Further studies are necessary to evaluate the cost–benefit ratios of different protocols in various environmental and management systems, particularly in resource-constrained regions. Additionally, emerging technologies like Letrozole-based protocols offer non-steroidal alternatives to traditional hormone treatments. These methods can potentially improve fertility outcomes while reducing dependence on estradiol, addressing both efficacy and regulatory concerns [77,78]. Continued research into such innovative approaches will be pivotal in advancing reproductive strategies for dairy cattle, ensuring both sustainability and productivity in the face of evolving challenges.

**Table 1:** Comparison of P/TAI in different synchronization protocols.

Protocol	P/TAI (%)	Reference
GnRH injection and CIDR Insertion Day 0 (Morning) + PGF2α injection, CIDR removal Day 5 (Morning) + Estrous detection Day 6 and 7 + GnRH injection and TAI Day 8	55.3	[79]
Double-Ovsynch (Pre-Ovsynch followed by Ovsynch-56 protocol)	56.3	[80]
PGF2α injection + GnRH injection 3–4 days later + GnRH injection and CIDR insertion 7 days later + PGF2α, eCG injection and CIDR removal 7 days later + GnRH injection 56 h later + TAI 16 h later	57.5	[81]
GnRH injection and CIDR insertion Day 0 + PGF2α injection, CIDR removal Day 5 (AM) + PGF2α injection 6 h later + GnRH injection Day 7 (PM) + TAI 56 h after CIDR removal	61.7	[22]
GnRH and CIDR Day 0 + PGF2α, CIDR removal Day 5 (AM) + PGF2α injection 6 h later + GnRH injection Day 7 (PM) + TAI 56 h after CIDR removal	63.6	[82]
Double-Ovsynch = Calving date as Day 0, GnRH injection Day 36 + PGF2α injection Day 46 + GnRH injection Day 49 followed by Ovsynch-GnRH injection Day 56 + PGF2α injection Day 63 + GnRH injection 56 h later + TAI 16 h later	69.5	[83]
EB injection + PGF2α injection Day 7 + GnRH injection Day 9 (EPG) during winter, Pregnancy Diagnosis on Day 60	68.75	[21]
PGF2α injection (Dinoprost) + EB injection 24 h later + TAI 24 h after EB	64.5	[73]
EB injection + GnRH injection Day 0 + PGF2α injection Day 7 + PGF2α injection Day 9	57.2	[68]
EB injection and P4 Device insertion Day 0 + GnRH injection Day 2 + PGF2α injection Day 7 + PGF2α, ECP injection and P4 Removal Day 9 + TAI Day11	40.5	[72]
EB, GnRH injection and P4 insertion Day 0 + PGF2α injection, P4 removal Day 7 + PGF2α, eCG and ECP injection Day 8 + Estrus and AI Day 10 + Pregnancy Diagnosis Day 30	57.4	[66]
EB, GnRH injection and P4 insertion Day 10 + PGF2α injection and US Day 3 + ECP, PGF2α injection, US, and P4 removal Day 2 + FTAI and US Day 0 + Pregnancy Diagnosis Day 32	60.9	[84]

US—ultrasound; ECP—estradiol cypionate; EPG—EB + PGF2α + GnRH.

## 3. Omics Integration in TAI

**Figure 3:**
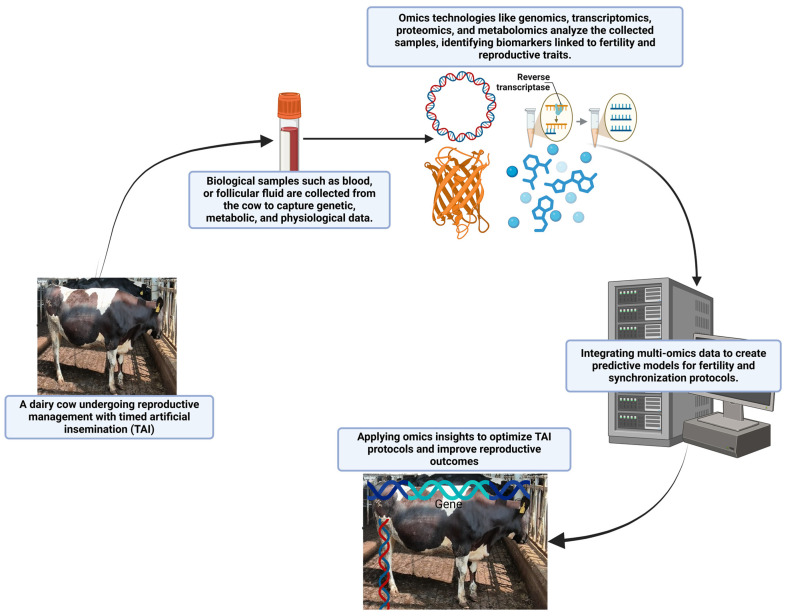
Integration of omics technologies into TAI in dairy cattle: A step-by-step representation of how biological sampling, omics analysis, data integration, and practical application work together to enhance reproductive efficiency and fertility management.

The integration of omics technologies such as genomics, transcriptomics, proteomics, and metabolomics into TAI has the potential to significantly improve reproductive outcomes in dairy cattle by offering deeper insights into the molecular and physiological processes underpinning fertility [28]. These technologies allow for a more individualized approach to breeding, which can enhance TAI protocols through precision breeding strategies, as shown in Figure 3 [85,86].

### 3.1. Genomics

Genomic selection has been a transformative tool in dairy cattle breeding, enabling farmers to select bulls and cows with superior reproductive traits [87,88,89]. Breeders can better predict which animals will respond favorably to TAI protocols by analyzing specific genomic markers associated with fertility. Recent studies have highlighted the role of genomic and epigenetic markers in predicting fertility outcomes. For example, Salilew-Wondim et al. (2023) [90] investigated how specific endometrial DNA methylation patterns influence fertility outcomes in dairy cows. They identified critical epigenetic markers, such as DNA methylome markers for fertility, and they found that genes like *LMTK3*, *PRDM16*, and *TMEM170A* showed a significant increase in methylation level that may be associated with an increase in pregnancy rate. These findings suggest that these epigenetic signatures could serve as valuable indicators of reproductive performance, demonstrating the potential to enhance fertility predictions and improve breeding strategies in dairy cattle by integrating genomic and epigenetic markers. Similarly, Sitko et al. (2024) [91] investigated the relationship between ovarian function, endocrine phenotypes, and genomic predictions of fertility potential in lactating dairy cows throughout the estrous cycle. The study revealed that cows with higher GDPRs exhibited better reproductive outcomes, higher progesterone concentrations, improved ovarian dynamics, and a greater likelihood of ovulation. Moreover, genomic analyses can identify cattle with traits linked to better conception rates, reduced calving intervals, and improved reproductive performance, underscoring the value of genomic markers in optimizing reproductive management strategies.

Further studies have highlighted the practical applications of genomic selection in improving fertility outcomes. Zolini et al. (2019) [92] investigated how genetic markers interact with human chorionic gonadotropin (hCG) treatments of TAI protocols to influence fertility outcomes in dairy cows, finding that genetic variations in genes like *COQ9*, *HSPA1L*, and *PGR* are associated with variations in pregnancy success rates. Specifically, it shows that genotype influences responses to hCG treatments, positively impacting fertility for certain SNP variations in *COQ9*, particularly in primiparous cows. Additionally, Sitko et al. (2024) [29] examined dairy cows’ reproductive physiological outcomes categorized by genomic merit for fertility, focusing on biomarkers, uterine health, endocrine status, and estrous behavior. They found that cows with higher genomic predicted transmitting ability values for fertility exhibited better reproductive traits, such as improved uterine health and higher progesterone levels during synchronization protocols. Although no significant differences were observed in metabolic indicators or estrous behavior across fertility groups, the study highlights the potential of genomic markers to inform breeding decisions and improve reproductive management. The findings suggest that understanding these physiological traits can enhance fertility predictions and outcomes in dairy cattle. Incorporating genomics into TAI allows for the optimization of breeding programs, particularly in herds with diverse genetic backgrounds, where traditional synchronization protocols may only be equally effective for some individuals.

The integration of genomics technologies with estrous synchronization protocols in dairy cattle offers a promising pathway to address the current limitations in TAI. Genomics can address the limitation of variability in cows’ responses to synchronization protocols due to differences in ovarian status, body condition, and genetic makeup [55,61] by identifying animals predisposed to higher fertility and favorable reproductive traits. Screening for specific genetic markers associated with hormonal responsiveness, such as GDPR, indicates higher fertility and responsiveness to synchronization protocols [29]. Breeders can select cows likely to respond well to protocols like GnRH-based or estradiol/P4-based protocols. This individualized approach minimizes the need for repeated insemination attempts and enhances the overall success of synchronization protocols. Additionally, research has shown that specific genomic markers, such as epigenetic signatures and genes related to fertility, can improve predictions of reproductive outcomes, making it possible to optimize TAI protocols for genetic diversity within herds [90,92]. Altogether, integrating genomic and epigenetic markers into reproductive management can revolutionize dairy cattle breeding by enabling precision-based TAI protocols, reducing repeated inseminations, and improving fertility outcomes. Omics technologies, particularly DNA genotyping, can help classify animals based on biological traits, allowing for targeted breeding strategies and optimized reproductive efficiency. These advancements also support management-based animal grouping, enhancing overall herd productivity. As research progresses, genomic tools will be essential in refining synchronization protocols, ensuring more efficient and effective breeding practices for diverse cattle populations.

### 3.2. Transcriptomics and Proteomics

Transcriptomic and proteomic analyses provide valuable insights into gene expression and protein activity during critical reproductive stages [93,94]. These technologies help identify biomarkers related to ovarian function, oocyte maturation, and embryo implantation, which are critical to the success of TAI protocols [95,96]. The transcriptomic profiling of endometrial and ovarian tissues has revealed gene expression patterns associated with estrous expression and ovulation timing, offering potential markers for predicting the optimal time for insemination. For example, a study by Ferraz et al. (2024) [97] evaluated several biomarkers for early pregnancy diagnosis in dairy cattle post-TAI, finding that the expression of interferon-stimulated genes *ISG15* and *RSAD2* in peripheral blood mononuclear cells were effective indicators of early pregnancy on Day 21. Additionally, plasma P4 on Day 21 and pregnancy-associated glycoproteins (PAGs) on Day 25 proved reliable, with PAGs being particularly effective for confirming pregnancy. This research demonstrates the potential of molecular markers to predict reproductive outcomes, enhancing TAI timing and success. Similarly, Ochoa et al. (2018) [98] examined the effects of PGF2α and prostaglandin E1 (PGE1) on gene expression within the CL of dairy cows, finding that PGF2α pulses induced significant changes in the expression of 955 genes associated with luteolytic transcriptomic changes, while simultaneous PGE1 treatment inhibited these changes and the gene expression induced by PGF2α, preserving CL function. Genes involved in immune response and prostaglandin synthesis were notably affected, illustrating that hormone-induced transcriptomic shifts in the CL are crucial for maintaining pregnancy. This highlights the role of hormone-induced transcriptomic shifts in supporting pregnancy.

Additionally, a study by Sharawy et al. (2023) [99] investigated the expression of genes related to angiogenesis and water transport during the Ovsynch protocol in Holstein dairy cows, finding the expression of critical genes like *VEGF*, *VEGFR2*, *AQP3*, and *AQP4* upregulated at specific times during the synchronization process, particularly around the PGF2α injection. These gene expression patterns are linked to ovarian function and can be potential biomarkers for predicting optimal insemination timing. Sousa et al. (2016) [100] investigated how eCG influences gene expression in the bovine CL, focusing on insulin signaling and angiogenesis pathways. Essential genes such as *INSR*, *IGF1*, and *GLUT4* were highlighted for their roles in luteal function and progesterone production. The study suggested that eCG treatment enhances the expression of these critical genes, which are vital for successful ovulation and estrus. Berisha et al. (2024) [101] further investigated the mRNA expression patterns of estrogen receptors (ESRRA and ESRRB) and *PGR* in bovine follicles during periovulation and CL formation. They found that the expression of these receptors was significantly reduced in preovulatory follicles during ovulation but increased in the newly formed CL. The study emphasized the role of gonadotropin surges in regulating these receptor expressions, which are crucial for ovulation and luteal function. Together, these findings demonstrate that transcriptomic profiling provides essential insights into the gene expression patterns and hormonal regulation of reproductive tissues, offering valuable biomarkers for timing insemination and enhancing fertility management in dairy cattle.

Proteomic analysis can complement transcriptomics by revealing the dynamic changes in protein levels related to hormone signaling during the estrous cycle, which can be used to optimize hormonal synchronization protocols [102,103]. For instance, Zachut et al. (2016) [104] characterized the protein composition in the follicular fluid (FF) of high- and low-fertility dairy cows to identify factors affecting reproductive success. The researchers identified 219 proteins, with notable differences in 8 proteins related to immune function and follicular development, i.e., *SERPINA1*, *TIMP2*, *ITIH1*, *HSPG2*, *C8A*, *COL1A2*, *F2*, and *IL1RAP*, in less fertile cows. These differences suggest that variations in protein levels in FF, linked to hormone signaling and follicle quality, may play a role in fertility and have implications for improving reproductive protocols through synchronization. Similarly, research by Chung et al. (2012) [105] compared the protein expression patterns in the CL of cyclic and pregnant cows, investigating how these changes support pregnancy. The study found 23 proteins, 6 of which were only found in pregnant CLs while the other 17 proteins were only found in cyclic CLs, including vimentin and MnSOD, which are essential for maintaining luteal function and are influenced by hormonal levels. These findings illustrate that specific proteins in the CL respond dynamically to hormonal changes, aiding pregnancy maintenance and providing potential markers for synchronization protocols. In summary, proteomic analyses offer valuable insights into the molecular factors governing reproductive function in dairy cows. Together with transcriptomics, they provide a powerful basis for identifying biomarkers that can improve fertility management, biomarkers for insemination timing, and enhance reproductive protocols in dairy production.

Transcriptomics allows for the real-time assessment of gene expression patterns associated with follicular development, ovulation, and early pregnancy [106]. Significant limitations of estradiol/P4-based protocols are variability in estrous expression and the difficulty in pinpointing optimal insemination timing [68,69]. Transcriptomics can address these by providing real-time information on gene expression associated with follicular readiness, ovulation, and early pregnancy, thereby helping to optimize the insemination timing for each cow. Similarly, proteomics provides additional practical applications by revealing proteins associated with reproductive processes that reflect the physiological status of the follicle or CL in response to hormone treatments [107]. In GnRH protocols, one limitation is inconsistency in hormonal responsiveness, which can affect synchronization outcomes [65]. Proteomics can mitigate this by monitoring specific protein markers that indicate the readiness of reproductive tissues, especially the CL, for hormone treatment [104] and by identifying key proteins, such as *SERPINA1* and *TIMP2*, that are associated with immune and hormonal responses in high-fertility cows. Breeders can assess whether a cow’s reproductive tissues are primed for conception by analyzing proteins in follicular fluid or CL tissue, such as those linked to immune function and hormonal response. Together, these technologies allow breeders to tailor synchronization protocols based on individual cows’ reproductive states, improving the accuracy and efficacy of TAI and boosting overall fertility outcomes. As research advances, integrating omics tools into reproductive management strategies will be essential for achieving sustainable and efficient dairy production.

### 3.3. Metabolomics

Metabolomics, which involves the comprehensive analysis of small molecules (metabolites) in biological samples, is a powerful tool for optimizing TAI protocols [108]. For example, the metabolic state of cows can influence their reproductive success, as imbalances in energy, nutrient levels, or metabolic pathways can affect oocyte quality and embryo viability. Gimeno et al. (2023) [109] identified biomarkers in the plasma of cows that predict successful pregnancies after embryo transfer, focusing on how metabolic profiles influence embryo viability. Biomarkers such as creatine, l-glutamine, and l-phenylalanine correlate with pregnancy success, particularly in recipients of frozen–thawed embryos [109]. These biomarkers, linked to the cow’s metabolic state, highlight that balanced energy and nutrient levels can enhance embryo viability and improve birth rates, emphasizing the importance of recipient metabolic health for embryo transfer success and suggesting these biomarkers may refine synchronization protocols and recipient selection [109]. Hessock et al. (2023) [110] examined the metabolite profile of preovulatory follicular fluid and how it responds to follicle development following estrous onset. The study found that the abundance of several metabolites, particularly those involved in amino acid and energy metabolism, significantly varied with time, with a notable increase in metabolites like pyruvate and glutamate during the early stages after estrous onset. These findings indicate that the metabolic state of the follicle environment is critical for preparing a viable oocyte, impacting embryo quality and fertility. The study concluded that metabolic balance within the follicular fluid is essential for reproductive success and provides potential markers to enhance synchronization timing.

Additionally, Chebel et al. (2020) [111] determined how GDPR relates to postpartum metabolic profiles and reproductive readiness in Holstein cows, focusing on the influence of metabolic state on fertility. They found that cows with higher GDPRs had more favorable metabolic profiles, including increased insulin-like growth factor-1 and glucose, which support energy balance, and decreased non-esterified fatty acids, indicating efficient energy use. These metabolic advantages were associated with earlier estrous onset and more robust estrus characteristics, suggesting that cows with balanced energy and nutrient levels experience improved reproductive success. The study concluded that selecting for GDPR can enhance fertility by promoting a stable metabolic state and reducing the reliance on hormonal synchronization for estrous management. Zhao et al. (2024) [112] analyzed metabolic and transcriptomic changes in yaks at induced estrus, examining how shifts in metabolic pathways support fertility. The study found significant metabolite changes in amino acid and lipid pathways across blood, urine, and follicular fluid, suggesting that these nutrients are crucial for oocyte viability and estrous readiness. These metabolic and genetic shifts highlight the importance of a balanced internal environment for successful reproduction. The findings suggest that specific metabolomic and transcriptomic profiles could guide synchronization protocols and improve reproductive success in yak breeding. Collectively, metabolomic analyses of blood, urine, or follicular fluid offer valuable insights into individual cows’ nutritional and metabolic status, allowing for adjustments in feeding regimes or supplementation strategies to ensure cows are in optimal condition for insemination and pregnancy. These findings suggest that metabolomics can play a vital role in improving reproductive success by guiding dietary and metabolic adjustments that support TAI protocols and enhance fertility outcomes.

Metabolomics enables breeders to monitor cows’ metabolic states, which are crucial for fertility and reproductive success. Metabolic imbalances can affect oocyte quality and embryo viability, influencing reproductive outcomes [113]. Analyzing metabolite profiles in blood, urine, or follicular fluid samples, breeders can detect imbalances and adjust cows’ diets or supplements as needed to create optimal conditions for conception. For instance, metabolites like creatine and l-glutamine correlate with higher embryo viability, suggesting that pre-insemination dietary adjustments could further support successful TAI [109]. Such tailored adjustments improve reproductive outcomes by ensuring cows are metabolically prepared for insemination and pregnancy. Collectively, metabolomics provides a powerful tool for tailoring TAI protocols, helping breeders improve fertility outcomes by ensuring cows are metabolically optimized for insemination and pregnancy. By identifying key metabolites and their roles in oocyte maturation, embryo viability, and estrous readiness, metabolomics enables breeders to make precise adjustments to cows’ diets and synchronization protocols. This individualized approach enhances the effectiveness of TAI protocols, ensuring cows are metabolically optimized for conception and improving overall fertility outcomes in dairy herds.

## 4. Challenges of Implementing Omics Technologies in TAI in Dairy Cattle

Although omics technologies promise to enhance TAI outcomes, their practical application remains challenging. Implementing these advanced technologies within TAI protocols in dairy cattle faces several obstacles. Research by Sitko et al. (2024) [91] highlighted that genetic variability affects reproductive responses, complicating the effectiveness of synchronization protocols. Additionally, the prevalence of atypical estrous cycles, particularly in cows with lower genomic merit, hinders successful outcomes [29]. This variability highlights the need for personalized approaches to synchronization that account for individual genetic and physiological differences. Ponce-Barajas et al. (2023) [93] highlighted the challenge of translating transcriptomic findings into practical applications for improving ovulation and fertility outcomes, addressing limitations in understanding the functional implications of gene expression changes in the CL related to progesterone production. Similarly, Ferraz et al. (2024) [97] also discussed the complexity of integrating multiple biomarkers to enhance early pregnancy diagnosis, emphasizing the need for the robust validation of these technologies in commercial settings. Furthermore, Zachut et al. (2016) [104] pointed out the difficulty in identifying specific proteins in follicular fluid that correlate with fertility, indicating a gap in understanding the biological roles of these proteins. Additionally, the variability in metabolomic profiles is influenced by follicular development, which complicates the establishment of consistent biomarkers for fertility prediction [110].

Moreover, implementing omics technologies in TAI is complicated by parity-based differences in reproductive physiology and hormonal responses. Omics technologies rely on molecular markers and predictive models, but the variability between nulliparous, primiparous, and multiparous cows complicates their standardization and accuracy. For example, nulliparous cows exhibit unique reproductive physiology and estrous cycle variability [114], making synchronization less predictable. Primiparous cows exhibit variability in reproductive responses due to genetic fertility merit and physiological differences, which complicate synchronization, reducing the effectiveness of TAI [4]. Additionally, multiparous cows have higher Anti-Müllerian Hormone (AMH) concentrations and superior fertility potential compared with nulliparous and primiparous cows [115], which can lead to differences in responses to hormonal treatments. These disparities might hinder the integration of omics technologies into TAI, necessitating more individualized synchronization strategies. These challenges underscore the necessity for further research and standardization in omics applications in TAI to effectively improve reproductive management in dairy herds. To address these obstacles, several priorities are identified:

1. Establishing consistent protocols for data collection, analysis, and interpretation to improve the reproducibility of findings.
2. Conducting large-scale studies to validate the reliability of identified biomarkers for predicting fertility and pregnancy outcomes.
3. Translating omics insights into actionable strategies that farmers can easily adopt, accounting for the variability in herd management systems.
4. Designing synchronization strategies tailored to individual cows’ genetic and physiological profiles to maximize TAI success.

Implementing omics technologies in TAI offers immense potential to revolutionize reproductive management in dairy cattle. However, challenges such as genetic variability, the complexity of translating research findings into practice, the lack of standardized methodologies, and parity-based differences in reproductive physiology and hormonal responses must be addressed. By overcoming these barriers, omics tools can become integral to enhancing synchronization protocols, improving fertility outcomes, and driving the efficiency of dairy operations.

## 5. Conclusions

TAI enhances reproductive efficiency in dairy cattle by optimizing insemination timing through controlled synchronization protocols. The integration of hormonal synchronization protocols, such as GnRH-based and estradiol/P4-based methods, has significantly enhanced fertility outcomes. However, success depends on several factors like cow physiology, insemination timing, and semen quality. Further advancements can be achieved by integrating omics technologies, including genomics, transcriptomics, and proteomics, to tailor protocols to individual cows’ genetic and physiological profiles. At the same time, metabolomics offers the potential for optimizing nutritional and metabolic status to improve reproductive health and TAI response. As dairy farming shifts toward precision-based management, TAI, combined with molecular insights, will play a crucial role in enhancing reproductive efficiency and herd productivity. Future research should optimize synchronization protocols for genetic variability and identify validated molecular biomarkers for early pregnancy detection while leveraging genomics alongside transcriptomics, proteomics, and metabolomics to refine fertility-related biological mechanisms, strengthening TAI’s effectiveness and contributing to sustainable dairy farming practices.

## Data Availability

All the data supporting the conclusions in this article have been presented in the manuscript.
